# Fluorescence-detected XAS with sub-second time resolution reveals new details about the redox activity of Pt/CeO_2_ catalyst

**DOI:** 10.1107/S1600577518005325

**Published:** 2018-05-30

**Authors:** Alexander A. Guda, Aram L. Bugaev, Rene Kopelent, Luca Braglia, Alexander V. Soldatov, Maarten Nachtegaal, Olga V. Safonova, Grigory Smolentsev

**Affiliations:** a The Smart Materials Research Center, Southern Federal University, Sladkova 174/28, Rostov-on-Don 344090, Russian Federation; b Department of Chemistry, NIS and CrisDi Interdepartmental Centres, asn INST Reference Center, University of Turin, Via P. Giuria 7, Turin 10125, Italy; c Paul Scherer Institute, Villigen 5232, Switzerland

**Keywords:** time-resolved XAS, fluorescence detection, heterogeneous catalysis, transient kinetics, CO oxidation, automotive catalysts, ceria, platinum.

## Abstract

A setup for fluorescence-detected X-ray absorption spectroscopy (XAS) with sub-second time resolution has been developed. This technique allows chemical speciation of low-concentrated materials embedded in highly absorbing matrices, which cannot be studied using transmission XAS. Using this setup, the reactivity of 1.5 wt% Pt/CeO_2_ catalyst was studied with 100 ms resolution during periodic cycling in CO- and oxygen-containing atmospheres in a plug-flow reactor. Measurements were performed at the Pt *L*_3_- and Ce *L*_3_-edges. The reactivity of platinum and cerium demonstrated a strong correlation. The oxidation of the catalyst starts on the ceria support helping the oxidation of platinum nanoparticles. The new time-resolved XAS setup can be applied to various systems, capable of reproducible cycling between different states triggered by gas atmosphere, light, temperature, *etc*. It opens up new perspectives for mechanistic studies on automotive catalysts, selective oxidation catalysts and photocatalysts.

## Abstract 2

A highly sensitive setup for measuring fluorescence-detected X-ray absorption spectroscopy with sub-second time resolution has been developed at the SuperXAS beamline at the Swiss Light Source. This opens new opportunities for understanding the dynamic structure of heterogeneous catalysts.

## 1. Introduction   

When performing a catalytic reaction, the atoms in the catalyst’s active site periodically change their local coordination and often the oxidation state. Detection of intermediate species and distinguishing them from inactive spectators allows the reaction mechanisms to be unravelled and helps the rational design of better catalysts. This type of research requires the development of highly sensitive spectroscopic methods (Weckhuysen, 2003 ▸; Meunier, 2010 ▸; Urakawa, 2016 ▸; Beale *et al.*, 2010 ▸) combined with advanced theory (Campbell, 2017 ▸). Spectroscopic methods with sub-second time resolution are needed to detect relevant intermediates (Gott & Oyama, 2009 ▸; Burch *et al.*, 2011 ▸; Kopelent *et al.*, 2015 ▸). Some important catalytic materials also operate under transient conditions. These are, for example, automotive exhaust catalysts (Trovarelli, 2002 ▸) and catalysts used in chemical looping processes (Guo *et al.*, 2014 ▸). Time-resolved spectroscopy is crucial to understand the functioning of these materials on the atomic scale (Yamamoto *et al.*, 2007 ▸; Nagai *et al.*, 2009 ▸; Newton *et al.*, 2010 ▸, 2012 ▸; Ferri *et al.*, 2014 ▸).

The use of time-resolved X-ray absorption spectroscopy (XAS) for *in situ* and *operando* studies of catalysts has increased significantly during the last few decades (Frenkel *et al.*, 2013 ▸; Newton & Dent, 2013 ▸). Several beamlines pioneering in this field (Frahm, 1988 ▸; Kaminaga *et al.*, 1981 ▸) are now complemented by new facilities equipped with oscillating channel-cut monochromators (Stötzel *et al.*, 2010 ▸; Fonda *et al.*, 2012 ▸; Nonaka *et al.*, 2012 ▸; Müller *et al.*, 2016 ▸), energy-dispersive (ED) polychromators (Pascarelli *et al.*, 2016 ▸; Kong *et al.*, 2012 ▸) and double-crystal monochromators performing rapid continuous energy scans (van Beek *et al.*, 2011 ▸; Nikitenko *et al.*, 2008 ▸; Dent *et al.*, 2009 ▸). For scanning monochromators, the acquisition time per 1000 eV range XAS spectrum can be varied depending on the setup from minutes to milliseconds. A time of 10 ms for high-quality XAS spectra was recently achieved by combining a novel oscillating channel-cut monochromator with fast ionization chambers (Müller *et al.*, 2016 ▸). ED polychromators operating under static conditions nowadays allow XAS spectra to be measured even faster, with sub-millisecond resolution (Pascarelli *et al.*, 2016 ▸; Pascarelli & Mathon, 2010 ▸).

However, XAS data acquisition with sub-second resolution is typically performed in transmission mode. This limits the application of time-resolved XAS for many types of industrially relevant catalytic materials which either contain low concentration of the element of interest or are supported on a highly X-ray absorbing matrix. Typical examples of such materials are metal-loaded ceria–zirconia catalysts used in the automotive industry and a large variety of oxide-supported catalysts containing less than 0.5% of the element of interest. Limitations can also arise from specific experimental conditions and reactor design not allowing for transmission detection. Therefore, the development of setups for measuring time-resolved fluorescence-detected XAS is of great importance for the catalysis community. Significant efforts have already been made in the development of such setups using PIPS silicon photodiodes combined with ED polychromators (Nagai *et al.*, 2009 ▸) and oscillating monochromators (Haumann *et al.*, 2005 ▸; Yao *et al.*, 2014 ▸; Zhang *et al.*, 2004 ▸; König *et al.*, 2014 ▸). However, XAS spectra obtained with silicon diodes contain strong background due to the lack of energy resolution. Therefore, these setups are not ideal for low-concentrated samples and detection of small spectral changes. Techniques for sub-second XAS acquisition using single-photon-counting fluorescence detectors are currently available for the 30 ns to 1 ms time range (Smolentsev *et al.*, 2014 ▸) and used for pump-and-probe studies of homogeneous photocatalysts excited by laser light. Using an X-ray emission spectrometer in the von Hamos geometry one can also measure X-ray absorption and emission spectra in fluorescence mode with sub-second time-resolution (Szlachetko *et al.*, 2012 ▸, 2013 ▸; Kopelent *et al.*, 2016 ▸). However, the efficiency of this detection system in terms of statistics is typically worse than that of standard single-photon-counting fluorescence detectors. This is due to the high-energy-resolution optics which selects only a small part of fluorescence photons and due to the low solid angle of currently available emission spectrometers. Therefore this detection system is also not ideal for low-concentrated samples.

In this work we developed a setup for time-resolved XAS acquisition with 20 ms to 1 s resolution. Using this method we revealed new details about the initial redox kinetics of a 1.5 wt% Pt/CeO_2_ catalyst under transient conditions. Noble metals on ceria-based supports are widely used by the automotive industry to reduce the content of CO, nitrous oxides and unreacted hydrocarbons in the car exhaust. These catalysts operate under fast oscillations of the gas composition (Trovarelli, 2002 ▸); therefore, it is important to unravel the role of each component in such catalysts with sub-second time-resolution. The low concentration of metal nanoparticles, as well as the highly absorbing nature of ceria, makes it difficult to gain a detailed understanding of the structure–function relationships. Until now, the dynamic structure of these catalysts was typically analysed by time-resolved high-energy X-ray diffraction and infrared spectroscopy (Newton *et al.*, 2010 ▸, 2012 ▸; Ferri *et al.*, 2014 ▸) which could not provide quantitative information on the oxidation state and local coordination of all elements of interest. Studies by time-resolved X-ray absorption methods on these catalysts are scarce (Yamamoto *et al.*, 2007 ▸; Nagai *et al.*, 2009 ▸; Marchionni *et al.*, 2016 ▸; Gibson *et al.*, 2017 ▸) due to the compromises between the optimal catalyst composition, the reaction conditions and the concentration of elements of interest in the X-ray beam. The setup developed in this contribution opens new opportunities for various catalyst compositions and allows for quantitative correlations between the reactivity of the noble metal and the support in plug-flow reactors down to 100 ms time resolution.

## 2. Materials and method   

### 2.1. X-ray source   

Ce *L*_3_ and Pt *L*_3_ XAS spectra were measured at the SuperXAS beamline (Abdala *et al.*, 2012 ▸) of the Swiss Light Source (SLS) at Paul Scherrer Institute, Switzerland. The incident beam was provided by a 2.9 T super-bend magnet. The SLS was running in the standard top-up mode at 2.4 GeV and with an average current of 400 mA. The Si surface of a collimating mirror at 2.5 mrad was used for harmonic rejection at the Ce *L*_3_-edge. For measurements at the Pt *L*_3_-edge we used the Rh-coated surface of this mirror. The energy was selected by a Si(111) channel-cut monochromator, which provides an energy resolution of Δ*E*/*E* = 2.0 × 10^−4^. Ce *L*_3_-edge static data were collected from 5620 eV to 5920 eV with a step of 1 eV near the edge and 3 eV in the EXAFS region. Pt *L*_3_-edge static data were collected from 11455 eV to 11755 eV with a step of 1 eV near the edge and 3 eV in the EXAFS region. The beam was focused down to 0.1 mm in the vertical direction and 0.5 mm in the horizontal direction by a Rh-coated toroidal mirror. A smaller vertical beam size was used to scan the catalyst bed along the flow direction. The photon flux obtained at the sample was about 3–5 × 10^11^ photons s^−1^ at the Ce *L*_3_-edge and 5–7 × 10^11^ photons s^−1^ at the Pt *L*_3_-edge.

### 2.2. Data acquisition system   

**Figure 1:**
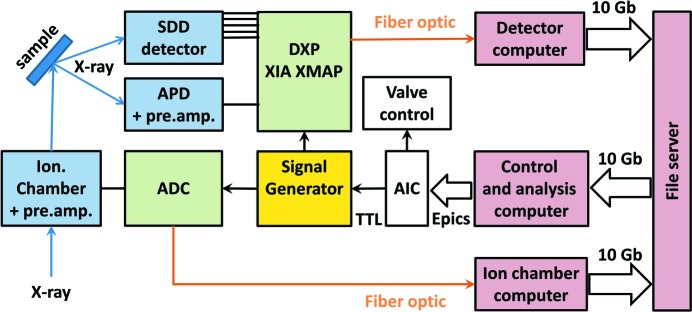
Scheme of the data acquisition system. SDD: five-element silicon drift detector; APD: avalanche photodiode; DXP: digital X-ray processor XIA XMAP operating in the multi-channel analyser mapping mode; ADC: analog-to-digital converter; AIC: analog input card. Blue boxes refer to the detectors; green boxes indicate the signal processing devices; red boxes correspond to the computers. The thick arrows show the communication lines between the devices with possible time delays, and the thin arrows refer to the delay-free connections. The control and analysis computer initiates an acquisition cycle, which is afterwards controlled only by a signal generator.

Fig. 1 ▸ shows a detailed scheme of the data acquisition (DAQ) system. In this DAQ, the fluorescence signal from the sample was detected by a five-element SGX silicon drift detector (SDD) and processed by XIA electronics (Digital X-ray Processor DXP-XMAP) operating in the multi-channel analyser mapping mode. In comparison, the pump-sequential-probe method with time-tagged photon counting uses a different, so-called ‘list mapping’, mode, allowing for 30 ns to 1 µs time resolution (Smolentsev *et al.*, 2014 ▸). The energy resolution of the detector was used to eliminate the elastic scattering and fluorescence signal from other elements.

**Figure 2:**
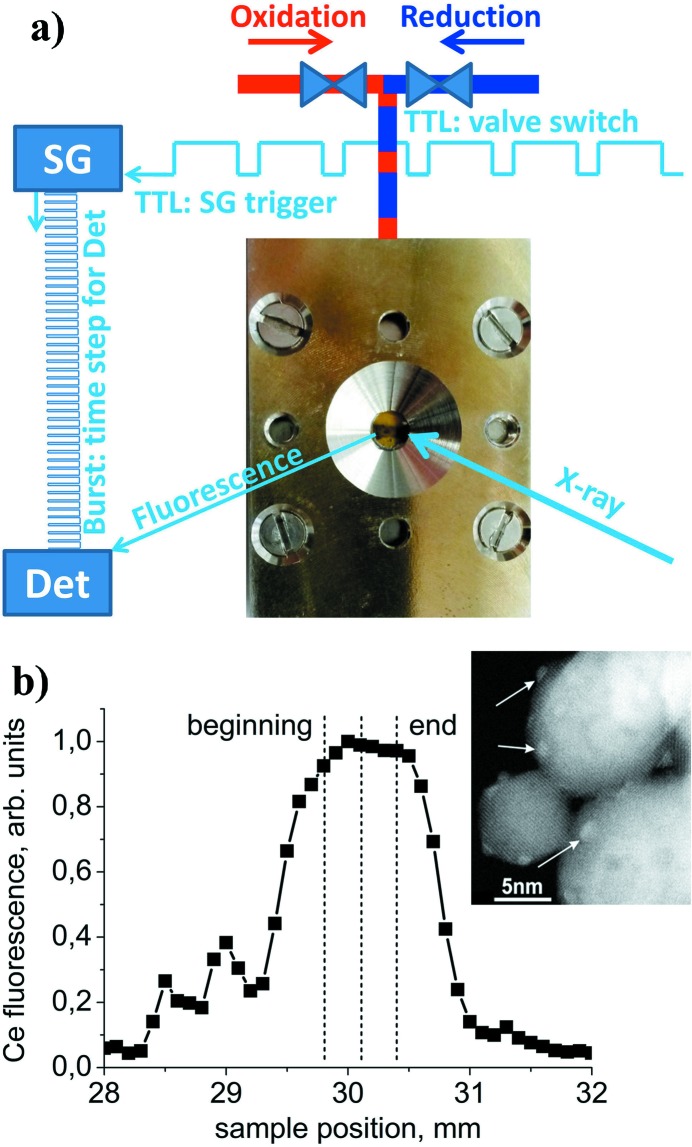
(*a*) Scheme of the experimental setup: a TTL signal initiates periodic switches between two gas flows in the cell and triggers the signal generator (SG) to produce a train of a fixed number of pulses that control the data acquisition system for the SDD detector (Det). (*b*) Vertical scan along the reactor cell using the Ce *L*α fluorescence signal showing the beginning and the end of the catalyst bed with respect to the direction of the gas flow. The inset shows a TEM image of 1.5 wt% Pt/CeO_2_ catalyst (Pt nanoparticles are indicated by arrows).

During the time-resolved experiments the gas composition in the reactor was periodically switched by two three-way switching valves (Parker, Series 9) which were triggered by a TTL signal generated by the beamline control system using an analog input card (AIC) (Hytec Electronics, Model VTB8204) (Fig. 2 ▸). The same TTL signal was sent simultaneously to a signal generator (Berkeley Nucleonics, Model 645, 50 MHz function/arbitrary waveform generator). Using the arrival of the TTL signal as the starting time, the signal generator produced a train of square pulses of equal duration and sent it to the gate of the XIA electronics. Each pulse triggered the collection of photons by the SDD detector. The width of each pulse determines the length of acquisition in each time point and, thus, the overall time-resolution of the experiment. After measuring the last time point the monochromator was moved to the next energy point and the time-resolved measurements, as described above, were repeated. To produce reliable data the reproducibility of the chemical processes in the catalytic cell is essential. To control it, we were always repeating transient experiments several times at one fixed energy (where the largest spectral changes were expected) before performing an experiment over the full energy range.

### 2.3. Sample and catalytic setup   

34 mg of 1.5 wt% Pt/CeO_2_ catalyst powder (Kopelent *et al.*, 2015 ▸) sieved to 100–150 µm grain size was placed into an *in situ* plug-flow reactor cell (Chiarello *et al.*, 2014 ▸) (Fig. 2 ▸) between two quartz wool plugs. High-surface-area ceria (85 m^2^ g^−1^) in the shape of truncated octahedral particles was prepared by a hydro­thermal method. Platinum nanoparticles of 1.2 (±0.2) nm diameter were supported on ceria by incipient wetness impregnation by tetraammine platinum(II) nitrate (Aldrich, 99.995%) followed by calcination in air (400°C, 4 h) and reduction in 5% H_2_ flow (300°C, 4 h). Further details of sample preparation and characterization are described by Kopelent *et al.* (2015 ▸). The reactor was connected to 5% CO in argon (CO 4.7 purity, Ar 5.0 purity), 21% O_2_ in argon (O_2_ 4.5 purity, Ar 5.0 purity) and argon (4.8 purity). The vertical orientation of the reactor enabled scanning of the reactor cell with a focused X-ray beam along the gas flow direction with 0.1 mm resolution. Mass flow controllers were producing two gas mixtures with the same total flow (50 ml min^−1^). One flow was directed to the cell while the other was going to the exhaust using two three-way switching valves (Fig. 2 ▸). A Pfeiffer Omnistar mass spectrometer was connected to the reactor outlet to analyse the reaction products. The reaction gases have not been pre­heated. However, we controlled the temperatures of the cell body and monitored the sample temperature at the beginning of the catalyst bed using a separate thermocouple. Working at relatively low temperatures, the difference between the cell and the sample temperatures did not exceed 1–2°C and changes in the catalyst temperature upon gas switching were also below 2°C. Thus, we do not expect a strong temperature gradient along the catalyst bed.

## 3. Results and discussion   

### 3.1. Performance of the setup   

The setup presented in this work uses a step-by-step energy scanning mode of the monochromator. It is designed under the assumption that the system under study can be reproducibly cycled between different states. In contrast, fast-scanning quick-XAS and static ED XAS setups can also be applied for studies of fast irreversible and non-reproducible processes, especially when the experiment can be performed in transmission mode on concentrated samples with optimized thickness. For low-concentrated samples this does not work due to insufficient statistics of the weaker fluorescence signal. Therefore, reproducibility and periodic cycling strategy (pump-and-probe strategy) are needed for low-concentrated systems to obtain high-quality time-resolved XAS spectra. As a step-by-step energy scanning does not bring additional limitations to the type of catalytic systems that can be studied, we have chosen this mode as it does not require any synchronization between the monochromator and the DAQ system.

The normalization to the incoming beam intensity (*I*_0_) is important for the detection of small spectral differences as in the top-up mode of the SLS storage ring the electron injections occur every three minutes changing the intensity by approximately 0.5%. We implemented two options for *I*_0_ normalization: (i) an avalanche photodiode (APD) detecting the signal from the elastic scattering of the incoming beam by air and registering it by an additional channel of the XIA electronics, and (ii) an ionization chamber placed before the sample and registering the transmission signal by a separate DAQ based on a fast ADC (National Instruments, Model PXIe-6366) card synchronized with the XIA DAQ by the TTL signal. The first option is simpler but the maximal count rate for one channel of the XIA is limited to ∼500000  counts s^−1^. If the concentration of the element of interest in the sample is low and the summed count rate for a selected fluorescence line is significantly lower than 500000 counts s^−1^, this method of normalization is sufficient. For concentrated samples (*e.g.* CeO_2_ support), when the count rate for the selected fluorescence line is much higher, *I*_0_ measured with this method will contribute to the noise level of the normalized spectra significantly. In this case, the use of the ionization chamber that measures *I*_0_ with good signal-to-noise ratio (∼10^−4^) is preferable. For example, when measuring time-resolved Ce *L*_3_ XAS spectra of a 0.5 wt% Pt/CeO_2_ catalyst with option (i) at 5726 eV we accumulated ∼200000 counts per time point with an APD detector and 120000 counts per time point from the Ce *L*_α_ fluorescence line. This gives the noise level of *I*_0_ of 0.3% and therefore this *I*_0_ signal could not be used to correct for 0.5% intensity jumps related to top-up mode of the synchrotron (see Fig. S1 of the supporting information). These intensity jumps were apparent in the data when the changes in the oxidation state of cerium during redox cycling of catalyst were less than 5%. With option (ii) for the *I*_0_ normalization, the noise level arising from the ionization chamber is 50 times smaller (0.006%).

The proposed DAQ scheme requires minimal communication between the control software and the detector, which allows any millisecond time delays or jitters to be avoided. The user defines the time that the sample spends in the different gas environments that are being periodically cycled and the overall time-resolution, which is then used to program the signal generator. In the multi-channel analyser mapping mode the full energy spectrum of each channel of the fluorescence detector is buffered at the XIA electronics for each time point, then streamed to the detector computer *via* an optical fiber connection, and finally saved to the file server in HDF5 format. A Python script reads the HDF5 files and processes the data in the energy and time dimensions for each detector channel to obtain kinetic and spectroscopic information. The script works with two threads: one controls the energy scan and acquisition, while the other is responsible for processing of the output files and visualization. There are several factors that can potentially limit the time resolution of the setup: (i) time resolution of the detection and the DAQ system, (ii) speed of the triggering, and (iii) speed of the data transfer and processing. The DXP card is rather fast: it has a 20 ns sampling time and thus provides a time resolution of the same order. SDD detectors are a bit slower: they have a resolution of about 1 µs, which is still sufficient for the present setup. Triggering has a nanosecond precision. The speed of data transfer and on-the-fly processing can be a limiting issue under some conditions. During preliminary tests with a slow and unstable 1 Gb network connection, the time resolution of the setup was limited to 100 ms. After a successful upgrade to a faster 10 Gb connection, the file transfer speed and the processing are not the limiting factors even for 20 ms resolution. As a possibility for further optimization, the DXP can be switched from the multi-channel analyser to the single-channel analyser mapping mode. In this case, the detector will only record the sum of the counts within a certain region of interest instead of the whole energy spectrum. This will significantly reduce the amount of data but will limit the possibilities for data reprocessing after the experiment.

In the current setup, the exchange of the gas atmosphere in the plug-flow reactor limits the time resolution of the whole system. We could detect changes in the catalyst state by XAS after 100 ms after the switch. The full replacement of the gas composition in the cell takes even longer; 90% gas exchange at 50 ml min^−1^ flow rate takes about 2 s. We demonstrated this by switching between 1% O_2_ in argon and pure argon and monitoring the decay of the *m*/*z* signal of 32 with mass spectrometry. These values can still be optimized by a further reduction of the dead volume in the cell and by increasing the flow rate. For some catalytic systems, alternative faster methods to trigger the changes in the reaction conditions can be used. For example, for photocatalytic systems, switching the light on and off can be used as a reaction trigger. With a LED-based source, the switching time can be reduced down to 1 µs, so that the speed of the reaction triggering will no longer be the limiting factor.

### 3.2. Quantitative analysis of time-resolved XAS spectra   

**Figure 3:**
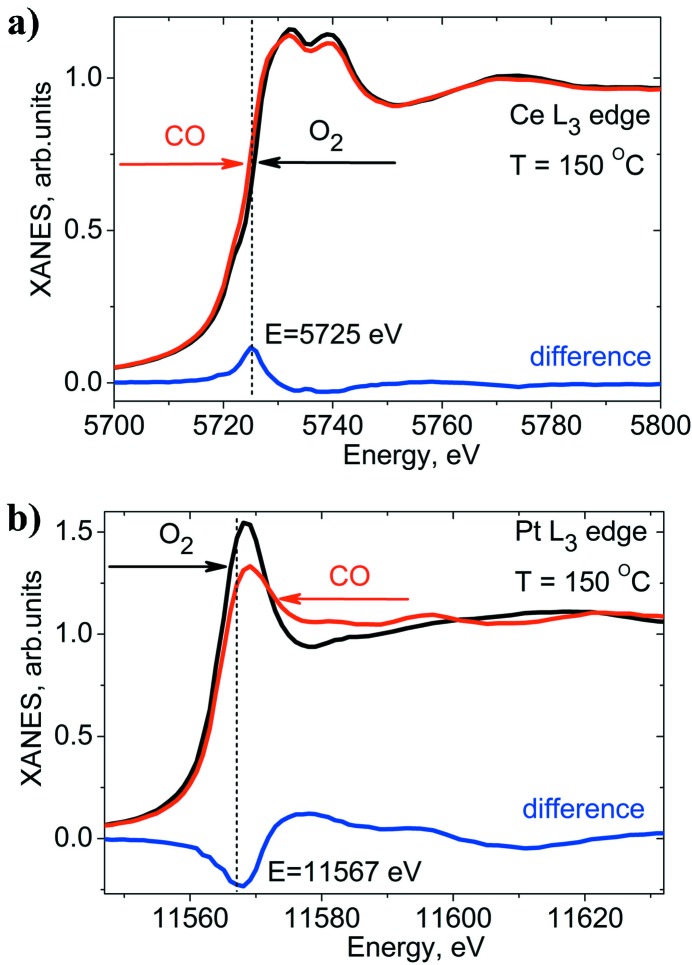
Static XAS spectra of 1.5 wt% Pt/CeO_2_ catalyst above the Ce *L*_3_ (*a*) and Pt *L*_3_ (*b*) edges measured under reducing (1% CO in argon) and oxidizing (4% O_2_ in argon) conditions at 150°C.

**Figure 4:**
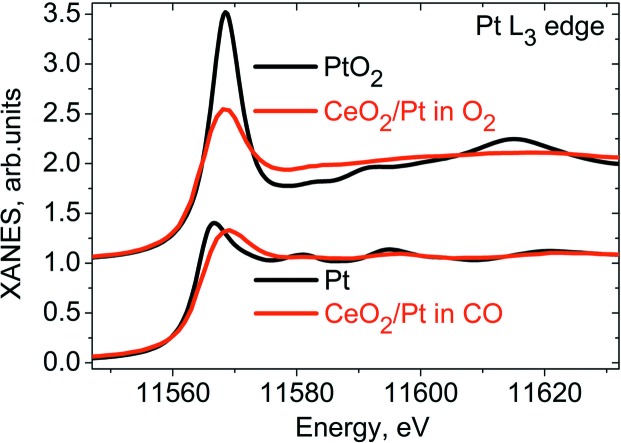
Pt *L*_3_ XAS spectra for 1.5 wt% Pt/CeO_2_ catalyst under reducing conditions (1% CO in argon at 150°C) and oxidizing conditions (4% O_2_ in argon at 150°C) are compared with the spectra of Pt foil and PtO_2_ references.

Fig. 3 ▸ shows the static Ce *L*_3_ and Pt *L*_3_ XAS spectra of the 1.5 wt% Pt/CeO_2_ catalyst in oxidizing (4% O_2_ in argon) and reducing (1% CO in argon) conditions at 150°C. Due to the high concentration of cerium in the catalyst, the Ce *L*_3_ XAS spectra demonstrate significant self-absorption. According to our previous studies (Safonova *et al.*, 2014 ▸), in 4% O_2_ at 150°C, the catalyst is fully oxidized to Ce^4+^ and when exposed to 1% CO it contains 11% Ce^3+^. This information allows for quantification of the Ce^3+^ concentration in transient experiments without self-absorption correction. Static XAS spectra at the Pt *L*_3_-edge also demonstrate significant changes as a function of the gas atmosphere composition. A comparison of the spectrum of the catalyst measured at 150°C in 4% O_2_ with the spectra of the platinum foil and PtO_2_ references (Fig. 4 ▸) suggests that the platinum nanoparticles (∼1.2 nm in diameter) on ceria are partially oxidized.

In 1% CO atmosphere, the Pt nanoparticles are reduced to the metallic state. The broad shape of the Pt *L*_3_-edge white line suggests CO adsorption at the platinum surface (Nikitenko *et al.*, 2008 ▸; Safonova *et al.*, 2006 ▸; Small *et al.*, 2012 ▸). The static Pt *L*_3_ XAS spectra of the catalyst in oxidizing (4% O_2_) and reducing (1% CO) atmospheres are almost independent of the temperature indicating that the static CO and oxygen coverage does not change significantly in the temperature range 25–150°C. Therefore, we preferred to quantify the changes in the coverage of platinum by oxygen rather than the oxidation state of platinum.

**Figure 5:**
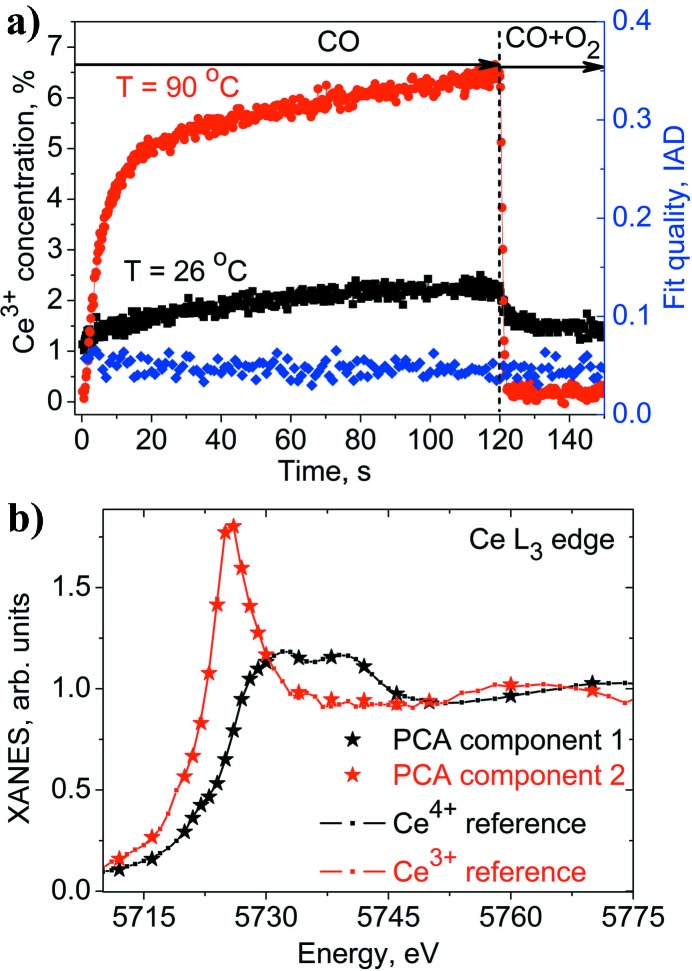
(*a*) Concentration of Ce^3+^ in 1.5 wt% Pt/CeO_2_ catalyst as a function of time during periodic cycling between 1% CO in argon and 1% CO + 4% O_2_ in argon at 26°C and 90°C. The fit quality for each time point was estimated as an integrated absolute difference (IAD) between the experimental spectrum and the sum of two PCA components. (*b*) PCA components are compared with Ce^3+^ and Ce^4+^ references calculated from data shown in Fig. 3 ▸.

By periodically changing the gas composition the catalyst was periodically reduced and oxidized. At each energy point of the full XAS spectrum these switches were performed and the signal was recorded with a 100 ms time resolution. The resulting reconstructed time-resolved Ce *L*_3_ XAS data were analysed quantitatively using the static XAS spectra plotted in Fig. 3 ▸. The 900 time-resolved XAS spectra (corresponding to a 180 s cycle with 100 ms time resolution) at 32 energy points (marked as stars in Fig. 5*b* ▸) were normalized to the edge jump of one and processed by using principal component analysis (PCA) and linear combination fit within the *FitIt* software package (Smolentsev & Soldatov, 2007 ▸; Smolentsev *et al.*, 2009 ▸). The spectra for the pure components were determined using all time-resolved XAS spectra plus the static XAS spectra of the same catalyst containing 0% and 11% of Ce^3+^. In addition, we applied the following constraints: the concentrations cannot be negative and the sum of all components in each spectrum should be equal to 100%.

PCA indicated two components in the spectra. Fig. 5(*a*) ▸ shows the results of the time-resolved quantification of the Ce^3+^ concentration in the 1.5 wt% Pt/CeO_2_ catalyst at two temperatures. The spectra of the Ce^3+^ and Ce^4+^ components determined by PCA are compared in Fig. 5(*b*) ▸ with the Ce^3+^ and Ce^4+^ components obtained from the static spectra containing 0% and 11% Ce^3+^ using a linear combination fit. The new method shows rather high sensitivity as it can detect changes in the oxidation state of cerium below 1% with 100 ms time resolution. If the element of interest changes only between two states, it is sufficient to probe the kinetics at only one fixed energy. The difference spectra in Fig. 3 ▸ demonstrate that the optimal energy for probing the oxidation state of cerium is 5725 eV, while for platinum it is 11567 eV.

### 3.3. Activity of the 1.5 wt% Pt/CeO_2_ catalyst during gas cycling   

**Figure 6:**
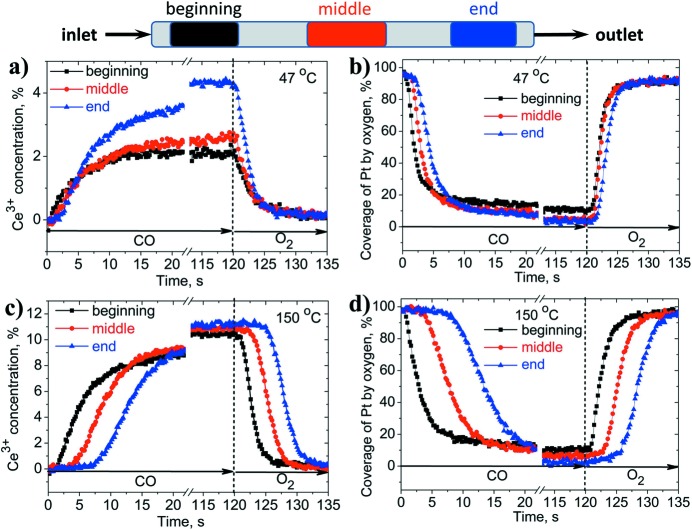
Concentration of Ce^3+^ and coverage of platinum nanoparticles by oxygen as a function of time upon switching between reducing (1% CO in argon) and oxidizing (4% O_2_ in argon) atmospheres at 47°C and 150°C in the beginning, in the middle and at the end of the reactor with respect to the inlet.

Fig. 6 ▸ shows the kinetics of the reversible evolution of the cerium and platinum states in the 1.5 wt% Pt/CeO_2_ catalyst during redox cycling in CO- and oxygen-containing atmospheres at 47°C and 150°C (the corresponding mass-spectrometer signal is shown in Fig. S2). For both elements, the kinetics strongly depends on the temperature and on the probed position in the reactor. This suggests that oxidation and reduction do not proceed simultaneously everywhere in the catalyst bed, especially at high temperatures, but rather propagate as a front through the reactor. Ceria is reduced stronger at high temperature than at low temperature. This reduction is completely reversible as the concentration of Ce^3+^ reduces to zero after exposure to oxygen. The maximal concentration of Ce^3+^ achieved after 120 s exposure to CO is independent of the position in the reactor and only at 47°C at the end of the catalyst bed we observed higher Ce^3+^ concentration than at the beginning. This can be explained by a small temperature gradient induced in the catalyst bed during the reduction in CO, which is an exothermal process.

When the catalyst approaches steady state in CO and oxygen, the state of platinum is almost independent of the temperature and the position in the catalyst bed. Thus, the equilibrium coverages of platinum by CO and oxygen are similar in the studied temperature range. We quantified the kinetics of cerium and platinum transformation at three positions in the plug-flow reactor using exponential decay functions (see §S2 of the supporting information). For each curve we determined the duration of the induction period and the time constants characterizing the rate of structural changes. The induction period corresponds to the time needed for the process to be initiated. The time constants allow the rates of the oxidation and reduction processes to be compared.

The induction periods for platinum and cerium are correlated. They are always longer at higher temperatures and at the end of the catalyst bed (Figs. 6 ▸ and S4). This indicates that the reaction front propagates slower in the catalyst bed at higher temperatures. At 150°C the redox dynamics are completely determined by the supply of the reactive gas (CO and oxygen) to the catalyst surface. At higher temperatures the ceria support is more reducible, more CO and oxygen molecules can be consumed; therefore, the propagation of the reaction front takes longer.

**Figure 7:**
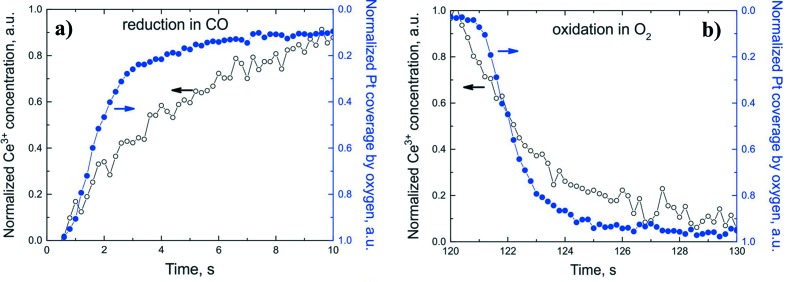
Correlation between the initial kinetics for the relative changes in Ce^3+^ concentration and platinum coverage by oxygen (normalized to the maximum) for two processes: reduction in 1% CO in argon (*a*) and oxidation in 4% O_2_ in argon (*b*) at 47°C in the beginning of the catalytic reactor.

In the low-temperature regime (47°C) the induction period is shorter and less dependent on the position in the catalyst bed (Fig. 6 ▸). Thus, the redox processes on the catalyst surface become slower and more important for the overall kinetics. Hence, the low-temperature (low conversion) regime is more appropriate for comparing the initial redox dynamics of the platinum nanoparticles and the ceria support. Fig. 7(*a*) ▸ demonstrates the redox activity of platinum and cerium in the low-temperature regime in the beginning of the catalyst bed. During reduction in CO, the induction periods for cerium and platinum are similar but the rate of platinum reduction is faster (smaller time constant). This suggests that the reduction starts almost at the same time on the platinum nanoparticles and the ceria support. Since oxidized ceria does not adsorb CO and would not reduce in the absence of platinum, the reduction of the catalyst should be initiated by CO spill-over on platinum nanoparticles. We cannot distinguish these steps, probably due to insufficient time resolution. Nevertheless, we see that platinum completes reduction faster than cerium. The reduction of the support is slower as it involves the diffusion of oxygen atoms on the surface and in the bulk of ceria. Table 1 ▸ shows a quantitative comparison for the fast time constants and the induction periods for three different positions in the reactor. In the middle and at the end of the catalyst bed, the induction periods are slightly longer and the time constants indicate slower kinetics.

Regarding the oxidation kinetics, interestingly, the induction period is reproducibly shorter for cerium than for platinum. Fig. 7(*b*) ▸ shows the oxidation kinetics for platinum and cerium at the beginning of the catalyst bed and Table 1 ▸ provides quantitative data for all three positions. This indicates that oxidation of the catalyst initiates on ceria. Since the surface of platinum nanoparticles is initially poisoned by CO, oxygen starts to react with Ce^3+^ sites on the support. The oxidation of the support helps the oxidation of the platinum nanoparticles providing the first active oxygen atoms to the metal–support interface. Previously, it was not clear from the literature (Vayssilov *et al.*, 2011 ▸; Grinter *et al.*, 2016 ▸; Christou & Efstathiou, 2007 ▸) whether the presence of platinum is always needed for the oxidation of the ceria-based supports. This oxidation process is similar to the reverse spillover of oxygen from ceria to platinum taking place in vacuum (Vayssilov *et al.*, 2011 ▸). We demonstrate that it can also take place under conditions relevant to the operation of exhaust catalysts. After initiation, the oxidation of platinum accelerates and ends up faster than that of cerium, as indicated by the lower time constant for platinum (Fig. 7*b* ▸ and Table 1 ▸). The slow overall kinetics of Ce^3+^ oxidation is explained by the slow diffusion of oxygen in the bulk structure of the support.

## 4. Conclusions   

We have developed a fluorescence-detected XAS-based setup for time-resolved studies of heterogeneous catalysts with a sub-second time resolution. This method offers new opportunities for kinetic studies of highly absorbing and low-concentrated materials, which cannot be investigated by using transmission-detected XAS. Using the new setup we revealed new details about the reactivity of a 1.5 wt% Pt/CeO_2_ catalyst in a plug-flow reactor during periodic redox cycling in CO and oxygen atmospheres. We demonstrate that the kinetics of oxidation and reduction of metal nanoparticles and the support are correlated and depend on the temperature and on the probed position in the reactor. We observed that the oxidation of the 1.5 wt% Pt/CeO_2_ catalyst starts on the ceria suggesting that ceria is more active than platinum during the oxidation cycle. The described setup can be applied to different systems which can be reproducibly cycled between different states using changes in gas atmosphere, light, temperature *etc*. with a time resolution up to 20 ms. The new setup opens new opportunities for mechanistic studies on a large variety of heterogeneous catalysts and photocatalysts.

## Supplementary Material

Supporting text, tables and figures. DOI: 10.1107/S1600577518005325/xj5013sup1.pdf

## Figures and Tables

**Table 1:** Time constants for the fast component and the induction period characterizing the initial kinetics of changes in the states of cerium and platinum in the 1.5 wt%/CeO_2_ catalyst during reduction in 1% CO in argon and oxidation in 4% O_2_ in argon at 47°C (see the supporting information for details)

	Reduction in CO			Oxidation in O_2_		
	Beginning	Middle	End	Beginning	Middle	End
Fast time constant (s)						
Ce	4.1	5.0	4.8	2.0	1.8	1.7
Pt	1.2	1.6	2.2	1.2	1.2	1.4

Induction period (s)						
Ce	0.5	1.2	2.3	0.4	0.6	0.7
Pt	0.7	1.5	2.4	1.2	1.4	2.1
