# Supplementary material for: Fluorescence-detected XAS with sub-second time resolution reveals new details about the redox activity of Pt/CeO2 catalyst
Source: J Synchrotron Radiat. 2018 May 30;25(Pt 4):989–97. doi: 10.1107/S1600577518005325 (PMC6038606; doi:10.1107/S1600577518005325)
Supplement: Supplementary file 1 [file s-25-00989-sup1.pdf]

JOURNAL OF SYNCHROTRON RADIATION

Volume 25 (2018)

Supporting information for article:

Fluorescence-detected XAS with sub-second time resolution reveals new details about the redox activity of Pt/CeO2 catalyst

Alexander A. Guda, Aram L. Bugaev, Rene Kopelent, Luca Braglia, Alexander V. Soldatov, Maarten Nachtegaal, Olga V. Safonova and Grigory Smolentsev

S1. Statistics of the I0 signal measured with avalanche photodiode (APD) and ionization chamber

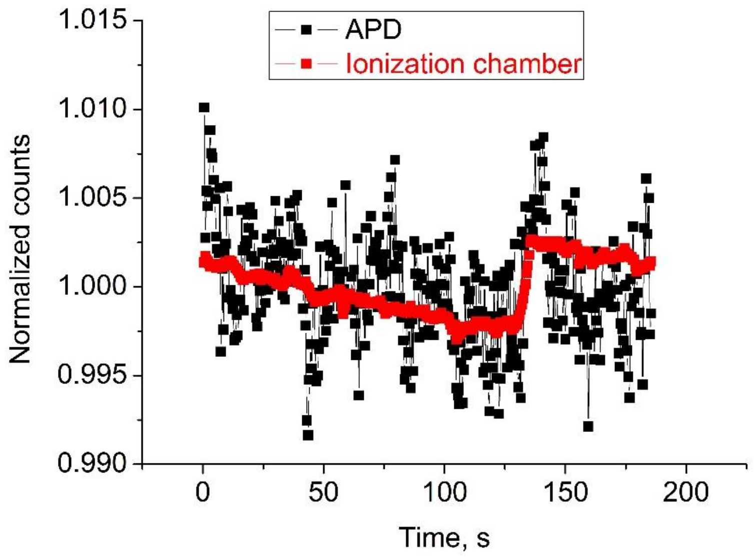

Figure S1 Normalized intensity of the incoming x-ray beam measured with APD and ionization chamber as a function of time. A step at 130s corresponds to the electron injection in the synchrotron ring operating in the top-up mode (occurring approx. every 3 minutes). Signal from the ionization chamber has much better statistics and is preferable for normalization.

S2. Mass-spectrometer data for the CO consumption and CO_2 formation for the studied Pt/CeO_2catalyst during experiment shown in Figure 6.

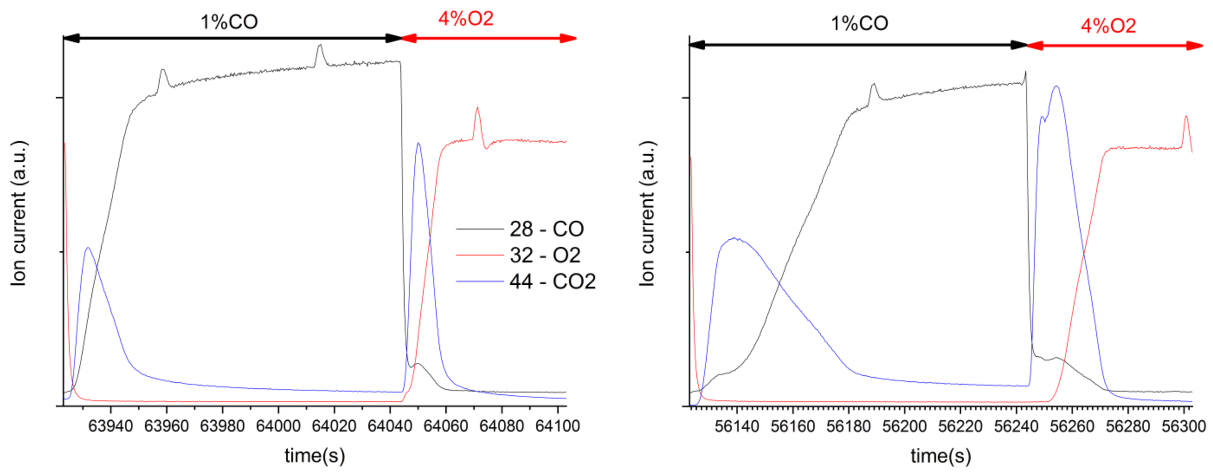

Figure S2 Evolution of the mass-spectrometer signals at the outlet on the cell for the transient experments shown in the Figure 6: 47 °C (left panel) and 150 °C (right panel). Ion currents registered for fragments with masses 28, 32 and 44 a.u. corresponding to CO, O_2 and CO_2 molecules were normalized to the signal of Ar with the mass of 40.

S3. Fitting of the time-resolved data for Ce L_3 edge.

To fit the changes in the Ce^{3+} concentration during the redox cycling of of 1.5 wt% Pt/CeO_2 catalyst in CO and oxygen containing atmospheres (shown in the Fig. 6 of the main text) we used a two-exponent function describing fast and slow components of the reaction process:

$$\boldsymbol{y(t)} = \begin{cases} \boldsymbol{Sc} \cdot \left((\boldsymbol{1} - \boldsymbol{C_0}) \cdot \left(\boldsymbol{1} - \boldsymbol{e}^{-\frac{t-t_0}{\tau_1}} \right) + \boldsymbol{C_0} \cdot \left(\boldsymbol{1} - \boldsymbol{e}^{-\frac{t-t_0}{\tau_2}} \right) \right), t \le 120 + t_1 \\ \boldsymbol{y}(\boldsymbol{120} + \boldsymbol{t_1}) \cdot \left((\boldsymbol{1} - \boldsymbol{C_1}) \cdot \boldsymbol{e}^{-\frac{t-t_1-120}{\tau_3}} + \boldsymbol{C_1} \cdot \boldsymbol{e}^{-\frac{t-t_1-120}{\tau_4}} \right), t > 120 + t_1 \end{cases}$$

The whole time range was split into two parts corresponding to reduction ($0<t<120$) and oxidation ($120<t<180$). At t = 0 s the gas flow was switched from 4% O_2 in argon to 1% CO in argon and at t = 120 s it was switched back. The following parameters were quantified:

Sc (Saturated concentration) - the asymptotic value of the concentration of reduced ceria at $t \to \infty$, %

$\boldsymbol{C_0}$ - fraction of the slow component in the fit for reduction

$\boldsymbol{C_1}$ - fraction of the slow component in the fit for oxidation

$\boldsymbol{t_0}$ - induction period of reduction, i.e. the delay time from the moment of the gas switch to the start of the exponential-like kinetics, s

$\boldsymbol{t_1}$ - induction period of oxidation, s

$\boldsymbol{\tau_1, \tau_2}$ - time constants for the fast and slow kinetics in the reduction process, s

$\boldsymbol{\tau_3, \tau_4}$ - time constants for the fast and slow kinetics in the oxidation process, s

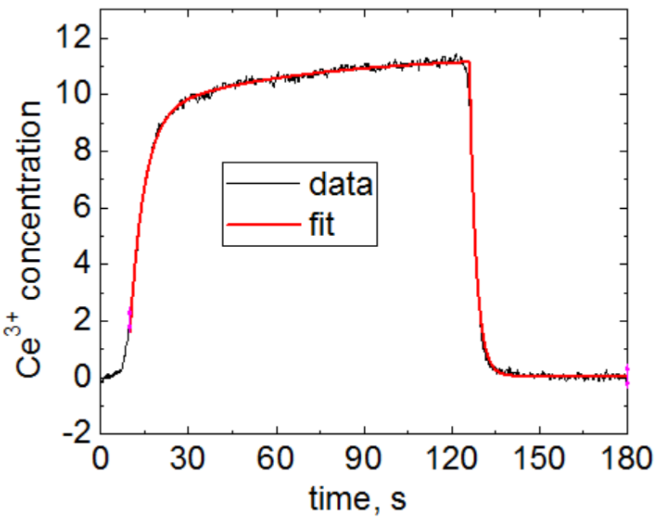

Figure S3 Typical changes in the Ce^{3+} concentration (%) during the redox cycling.

Table S1 Kinetic parameters for the evolution of the Ce3+ concentration during the redox cycling of 1.5 wt% Pt/CeO2 catalyst at 47 °C in the beginning, in the middle, and at the end of the catalyst bed with respect to the reactor inlet (z corresponds to distance in millimeters from the reactor inlet).

	z=0	z=0.3	z=0.6	z=0	z=0.3	z=0.6
	47 ˚C reduction			47 ˚C oxidation		
Saturated Ce^{3+} concentration (%)	2.1	2.5	4.5	-	-	-
Induction period (s)	0.5	1.2	2.3	0.4	0.6	0.7
Fast time constant (s)	4.1	5.0	4.8	2.0	1.8	1.7
Slow time constant (s)	-[a]	29	55	9	12	10

[a]Values not contributing to the fit

Table S2 Kinetic parameters for the evolution of the Ce^{3+} concentration during the redox cycling of 1.5 wt% Pt/CeO_2 catalyst at 150 ˚C in the beginning, in the middle, and at the end of the catalyst bed with respect to the reactor inlet (z corresponds to distance in millimeters from the reactor inlet)

	z=0	z=0.3	z=0.6	z=0	z=0.3	z=0.6
	150 ˚C reduction			150 ˚C oxidation		
Saturated Ce^{3+} concentration (%)	10.8	11.0	11.4	-	-	-
Induction period (s)	1.0	5.3	9.1	1.5	3.6	6.1
Fast time constant (s)	5.0	4.3	4.9	1.5	2.1	2.3
Slow time constant (s)	63	53	53	26	-[a]	-

[a]Values not contributing to the fit

S4. Fitting of the time-resolved data for Pt L_3 edge.

To fit the changes in the platinum coverage by oxygen during the redox cycling of Pt/CeO_2 catalyst in CO and oxygen containing atmospheres (shown in the Fig. 6 of the main text) we used a two-exponent function describing fast and slow components of the reaction process:

$$y(t) = \begin{cases} 100 - Sc \cdot \left((1 - C_0) \cdot \left(1 - e^{-\frac{t-t_0}{\tau_1}} \right) + C_0 \cdot \left(1 - e^{-\frac{t-t_0}{\tau_2}} \right) \right), t \le 120 + t_1 \\ y(120 + t_1) \cdot \left((1 - C_1) \cdot e^{-\frac{t-t_1-120}{\tau_3}} + C_1 \cdot e^{-\frac{t-t_1-120}{\tau_4}} \right), t > 120 + t_1 \end{cases}$$

The whole time range was split into two parts corresponding to reduction (0<t<120) and oxidation (120<t<180). At t = 0 s the gas flow was switched from 4% O_2 in argon to 1% CO in argon and at t = 120 s it was switched back. The following parameters were quantified:

Sc – amplitude of changes in platinum coverage by oxygen %

C_0 - fraction of the slow component in the fit for reduction

C_1 - fraction of the slow component in the fit for oxidation

t_0 - induction period of reduction, i.e. the delay time from the moment of the gas switch to the start of the exponential-like kinetics, s

t_1 - induction period of oxidation, s

τ_1, τ_2 - time constants for the fast and slow kinetics in the reduction process, s

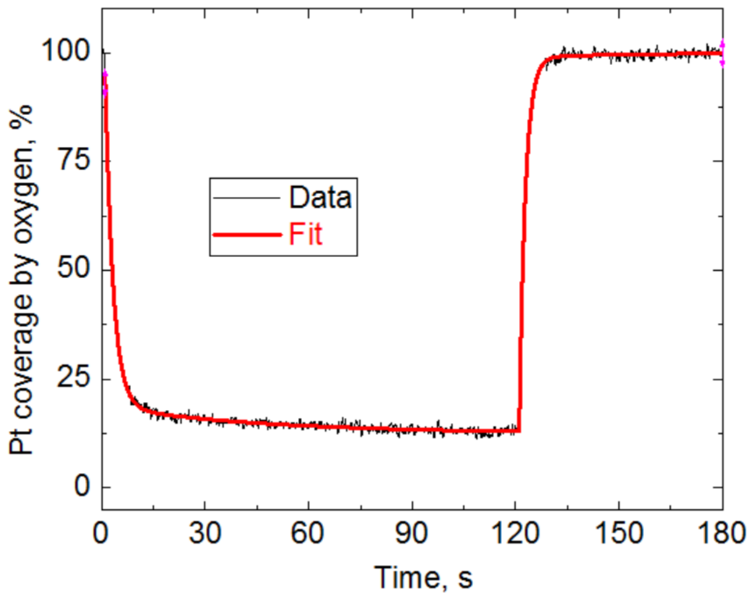

Figure S4 Typical changes in the coverage of platinum by oxygen (%) during the redox cycling.

Table S3 Kinetic parameters for the evolution of the platinum coverage by oxygen during the redox cycling of 1.5 wt% Pt/CeO_2 catalyst at 47 ˚C in the beginning, in the middle, and at the end of the catalyst bed with respect to the reactor inlet (z corresponds to distance in millimeters from the reactor inlet).

	z=0	z=0.3	z=0.6	z=0	z=0.3	z=0.6
	47 ˚C reduction			47 ˚C oxidation		
Induction period (s)	0.7	1.5	2.4	1.2	1.4	2.1
Fast time constant (s)	1.2	1.6	2.1	1.2	1.2	1.4
Slow time constant (s)	15	29	28	-[a]	-	-

[a]Values not contributing to the fit

Table S4 Kinetic parameters for the evolution of the platinum coverage by oxygen during the redox cycling at 150 ˚C of 1.5 wt% Pt/CeO_2 catalyst in the beginning, in the middle, and at the end of the catalyst bed with respect to the reactor inlet (z corresponds to distance in millimeters from the reactor inlet).

	z=0	z=0.4	z=0.8	z=0	z=0.4	z=0.8
	150 ˚C reduction			150 ˚C oxidation		
Induction period (s)	0.8	5.1	10.4	1.1	3.6	6.8
Fast time constant (s)	2.3	3.2	4.0	1.8	2.1	2.3
Slow time constant (s)	45	35	52	-[a]	-	-

[a]Values not contributing to the fit

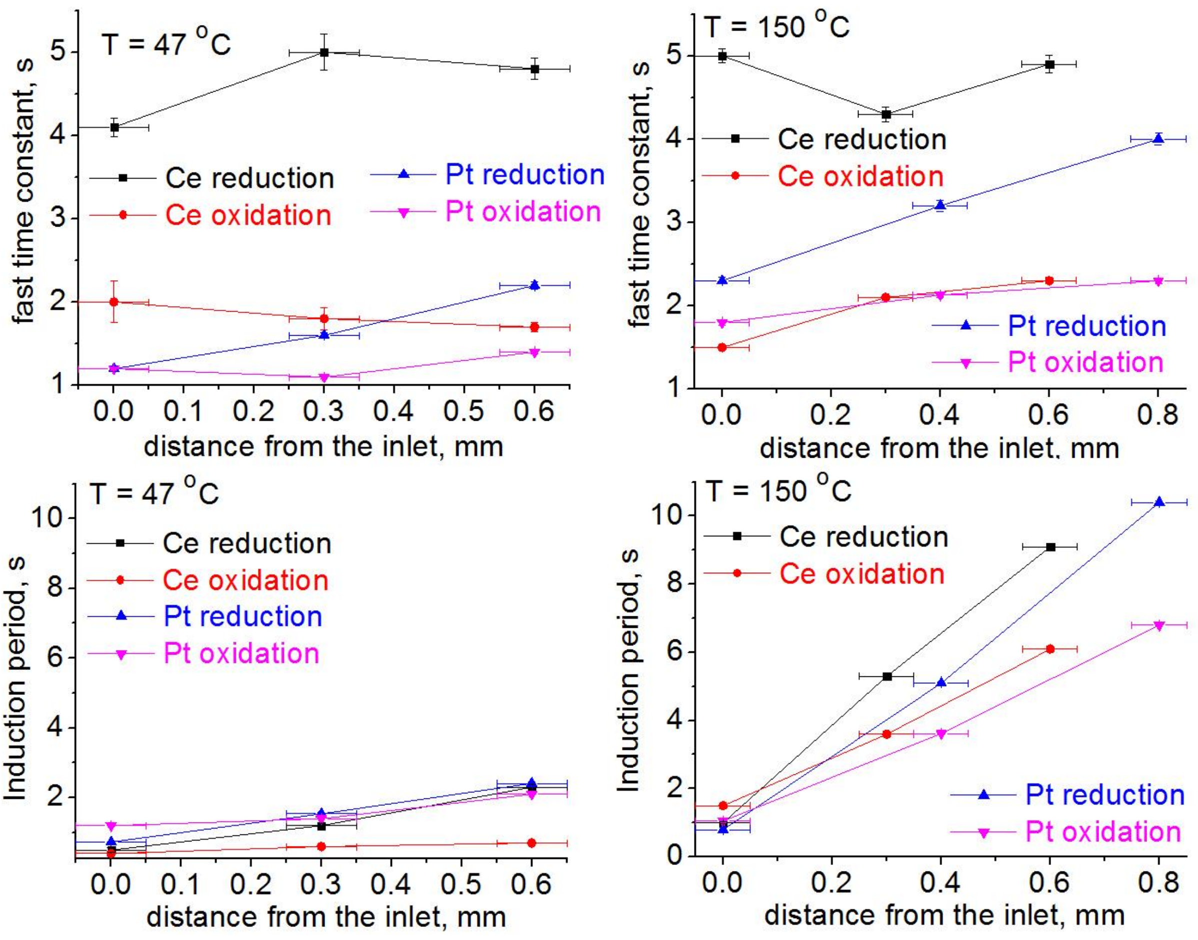

Figure S5 Evolution of the kinetic parameters shown in Tables S1-S4 as a function of distance from the reactor inlet (z). The error bars for the x-axis correspond to a half width of the X-ray beam in the vertical direction. The error bars for the y-axis were estimated from the fitting procedure. The error bars for the induction period are smaller than the symbols.

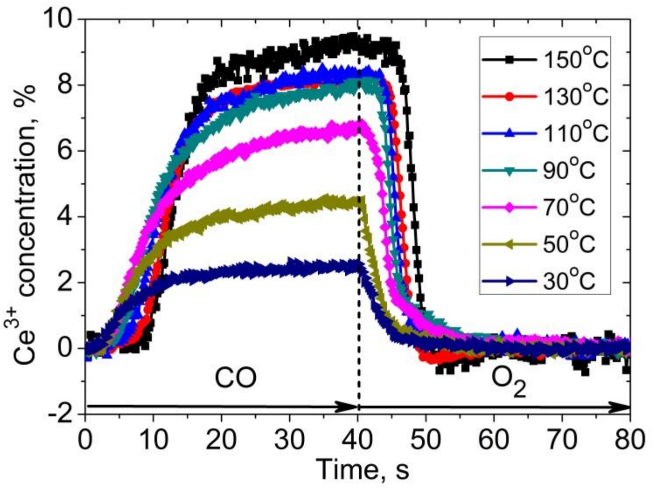

Figure S6 Evolution of the Ce^{3+} concentration as a function of time at different temperatures at a fixed position (z) in the reactor.

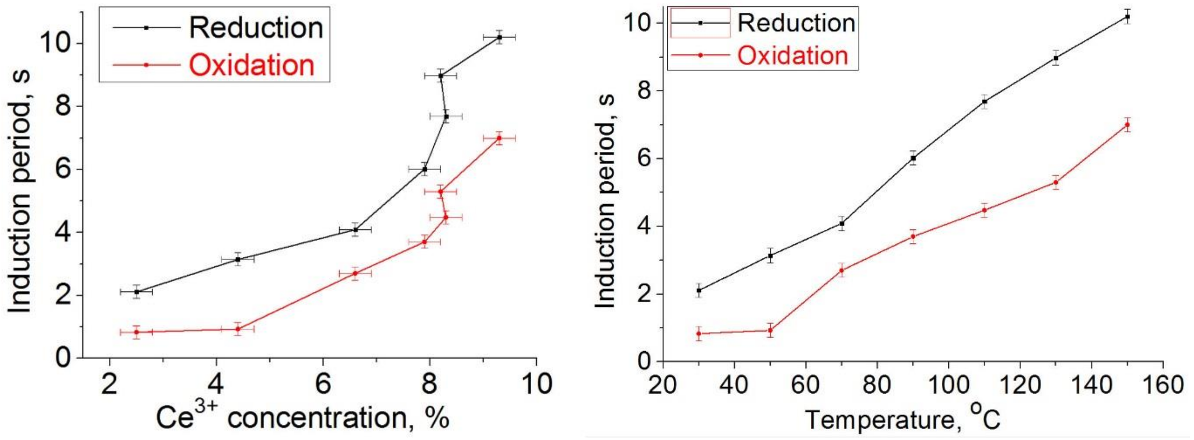

Figure S7 The induction period for Ce^{3+} formation (reduction) and Ce^{3+} decay (oxidation) as a function of the maximal Ce^{3+} concentration and the temperature. The error bars were estimated from the fitting procedure. Larger errors for Ce^{3+} concentration in comparison to Figure S5 are related to shorter time of experiments.

S5. Quality and details of all fits

Ce^{3+} concentration	T = 47 °C	z = 0.0 mm from the inlet

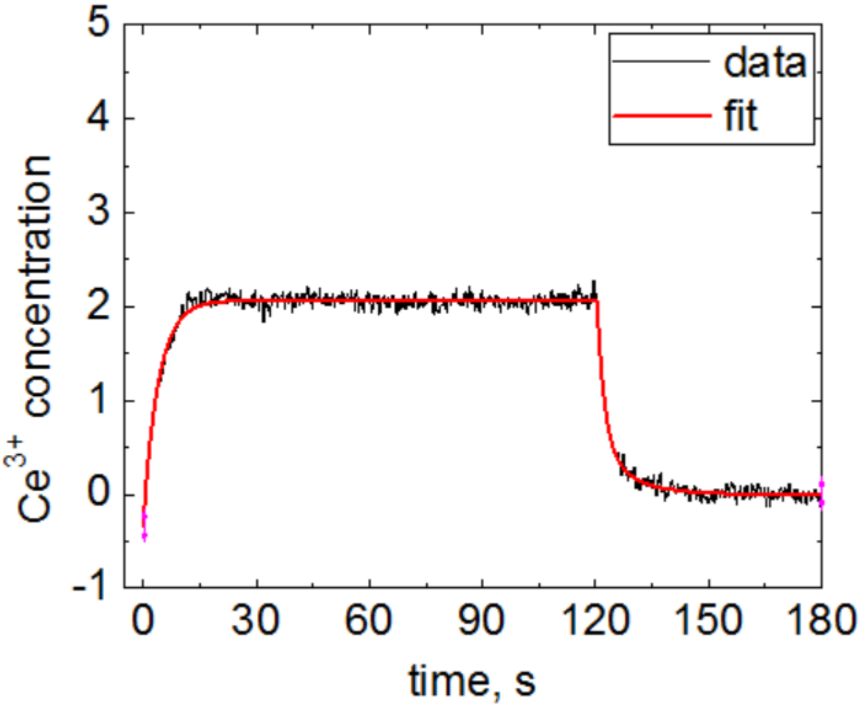

Parameter	Value	Error
Sc	2.067	0.004
C_0	0.000	0.002
t_0	0.497	0.070
τ_1	4.072	0.106
τ_2	0.025	4.573
C_1	0.255	0.071
t_1	0.385	0.064
τ_3	2.032	0.246
τ_4	8.517	1.505
Fit index, χ	0.007	

**Ce^{3+} concentration**	**T = 47 °C**	**z = 0.3 mm from the inlet**

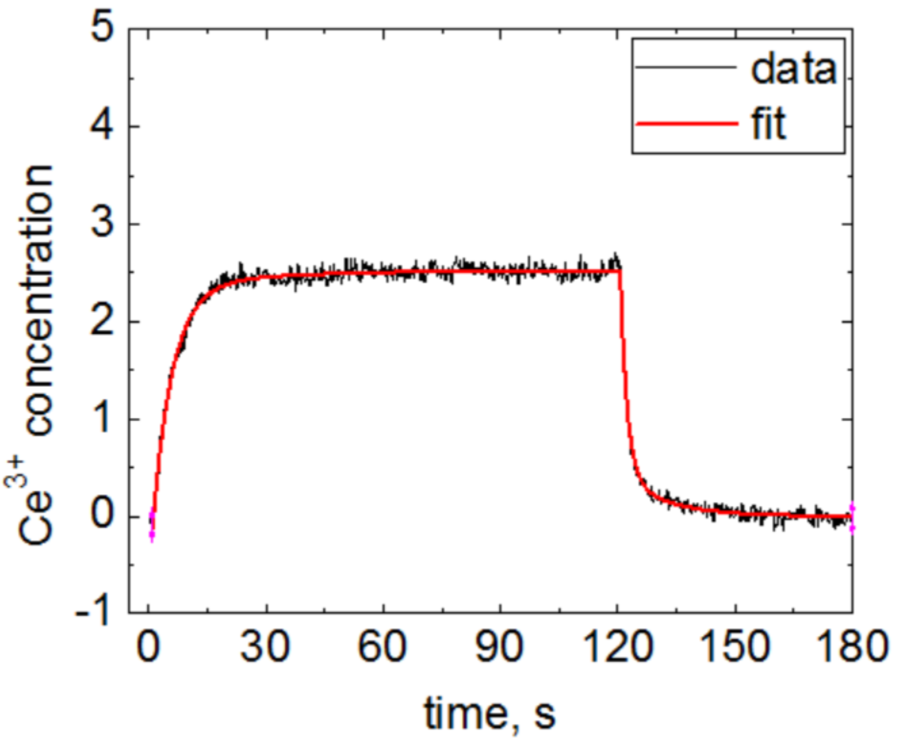

**Parameter**	**Value**	**Error**
Sc	2.528	0.011
C_0	0.070	0.026
t_0	1.231	0.063
τ_1	5.015	0.221
τ_2	28.53	12.79
C_1	0.169	0.027
t_1	0.600	0.054
τ_3	1.772	0.127
τ_4	11.59	1.521
Fit index, χ	0.006	

Ce^{3+} concentration	T = 47 °C	z = 0.6 mm from the inlet

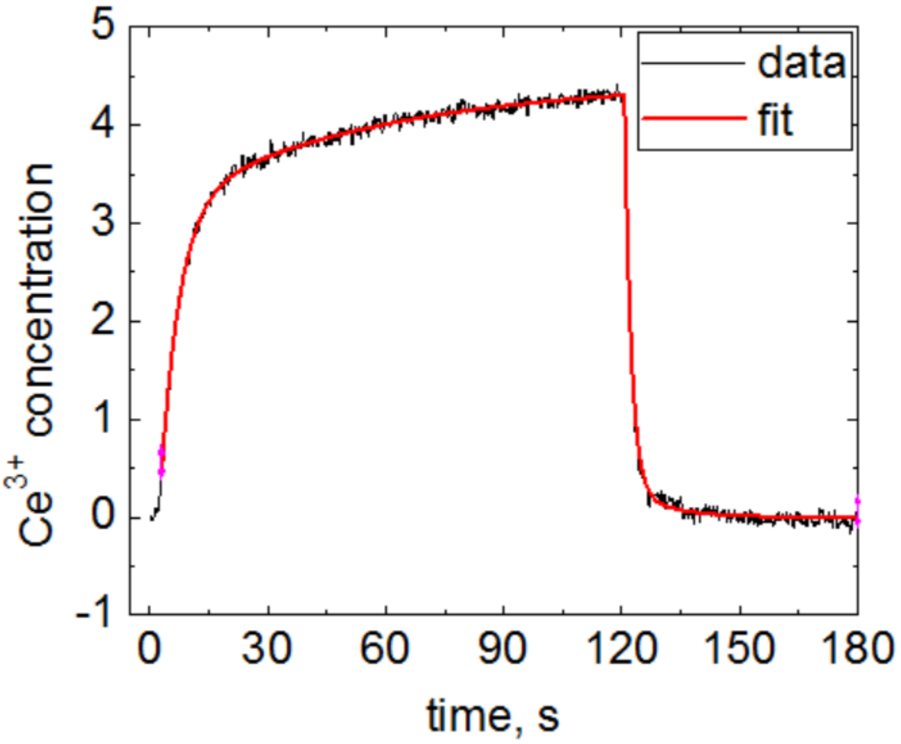

Parameter	Value	Error
Sc	4.452	0.029
C_0	0.285	0.005
t_0	2.319	0.060
τ_1	4.810	0.130
τ_2	54.78	3.905
C_1	0.069	0.017
t_1	0.697	0.023
τ_3	1.725	0.058
τ_4	10.24	1.904
Fit index, χ	0.005	

Ce^{3+} concentration	T = 150 °C	z = 0 mm from the inlet
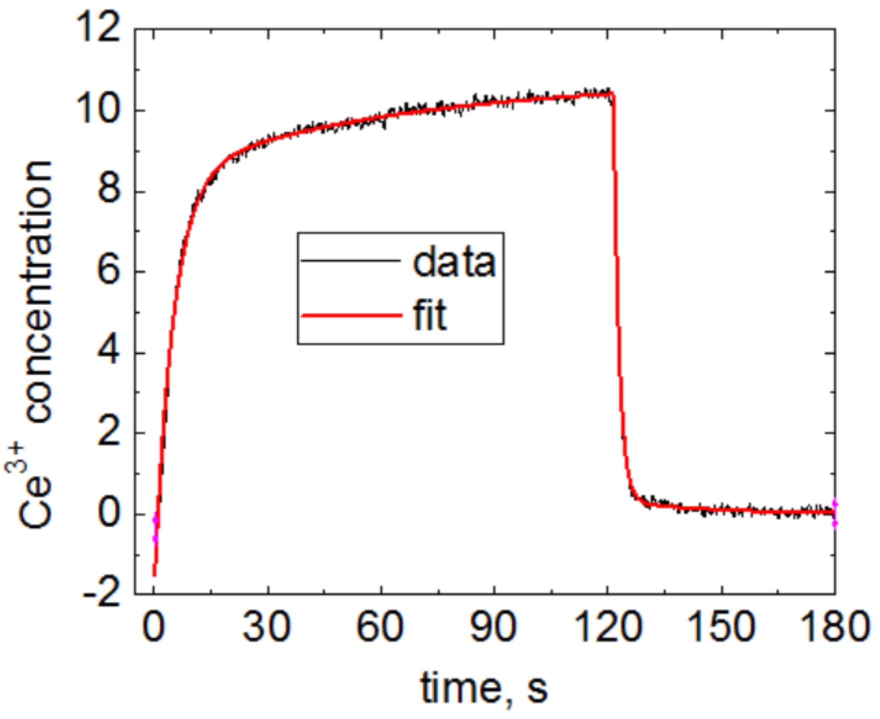		
**Parameter**	**Value**	**Error**
Sc	10.76	0.080
C_0	0.219	0.004
t_0	1.015	0.027
τ_1	5.012	0.084
τ_2	62.97	5.929
C_1	0.032	0.005
t_1	1.477	0.019
τ_3	1.497	0.033
τ_4	26.45	4.861
Fit index, χ	0.027	

Ce^{3+} concentration	T = 150 °C	z = 0.3 mm from the inlet

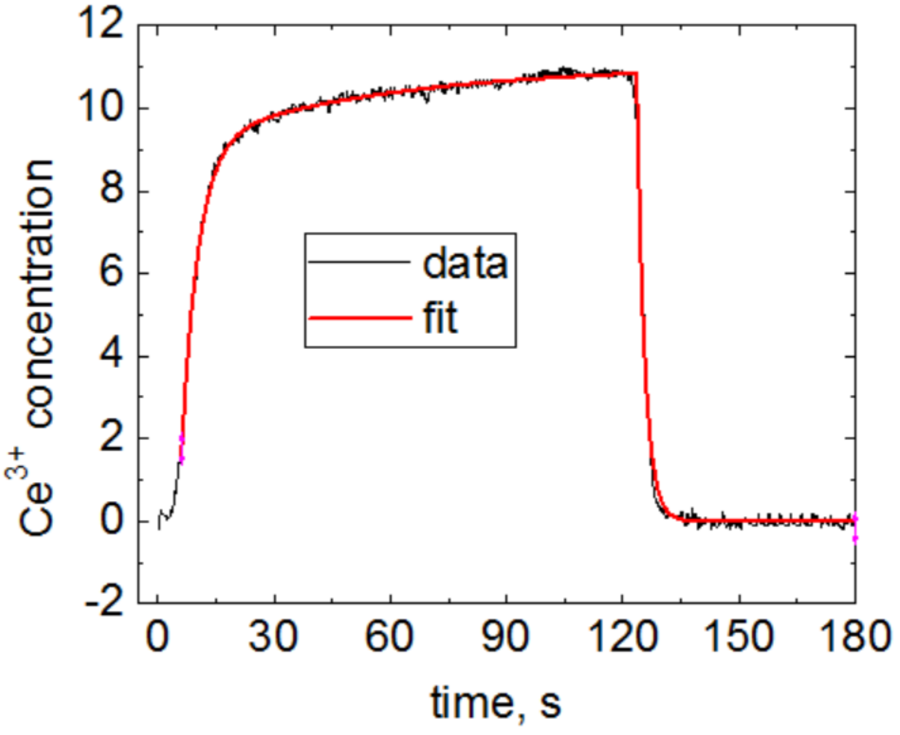

Parameter	Value	Error
Sc	11.04	0.057
C_0	0.173	0.004
t_0	5.255	0.048
τ_1	4.346	0.094
τ_2	52.50	5.073
C_1	0.000	--
t_1	3.568	0.018
τ_3	2.093	0.026
τ_4	0.000	0.000
Fit index, χ	0.025	

**Ce^{3+} concentration**	**T = 150 °C**	**z = 0.6 mm from the inlet**

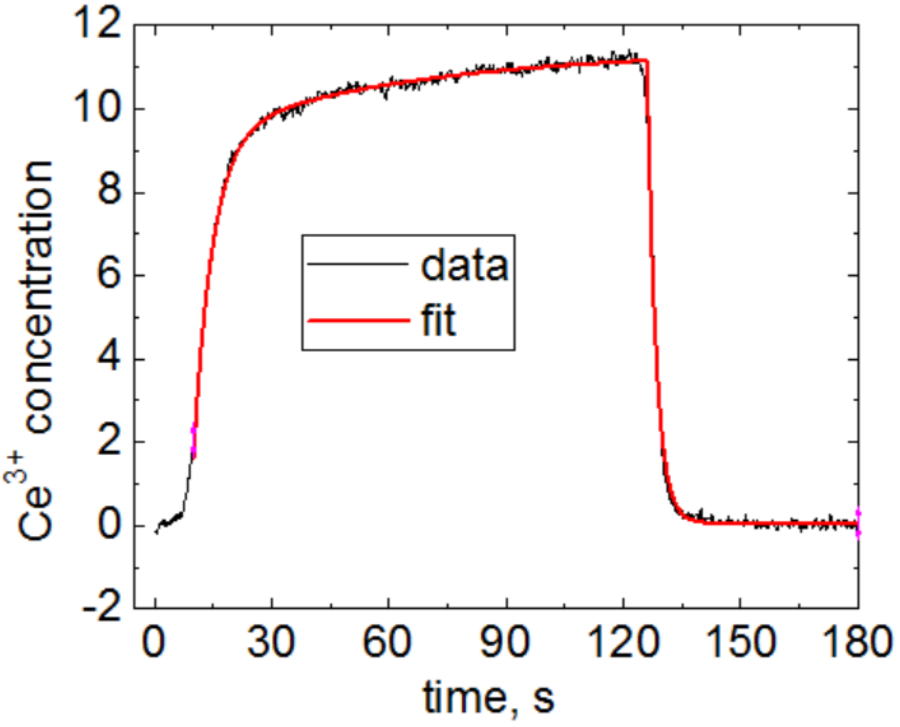

**Parameter**	**Value**	**Error**
Sc	11.39	0.066
C_0	0.185	0.005
t_0	9.092	0.053
τ_1	4.869	0.110
τ_2	53.43	5.411
C_1	0.006	0.003
t_1	6.081	0.021
τ_3	2.289	0.036
τ_4	90.81	138.9
Fit index, χ	0.027	

**Coverage of platinum by oxygen, %**	**T = 47 °C**	**z = 0.0 mm from the inlet**
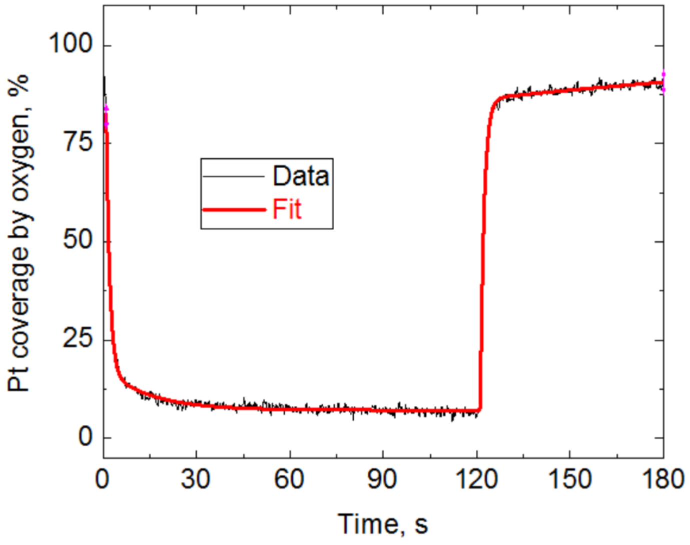		
**Parameter**	**Value**	**Error**
C_0	0.111	0.005
t_0	0.730	0.018
τ_1	1.148	0.029
τ_2	14.47	0.854
C_1	0.148	0.002
t_1	1.168	0.012
τ_3	1.203	0.020
τ_4	155.8	9.070
Fit index, χ	1.144	

**Coverage of platinum by oxygen, %**	**T = 47 °C**	**z = 0.3 mm from the inlet**

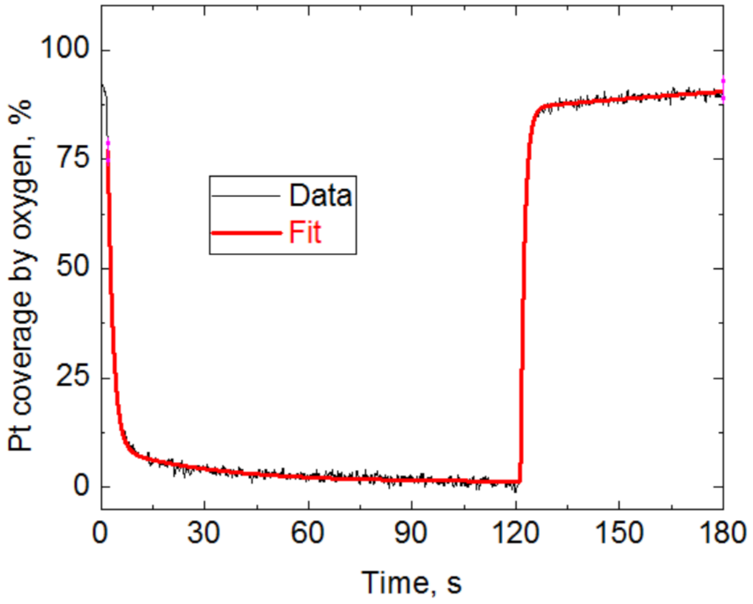

**Parameter**	**Value**	**Error**
C_0	0.081	0.003
t_0	1.543	0.022
τ_1	1.566	0.030
τ_2	28.89	2.107
C_1	0.135	0.002
t_1	1.377	0.011
τ_3	1.171	0.018
τ_4	178.8	11.86
Fit index, χ	1.110	

Coverage of platinum by oxygen, %	T = 47 °C	z = 0.6 mm from the inlet
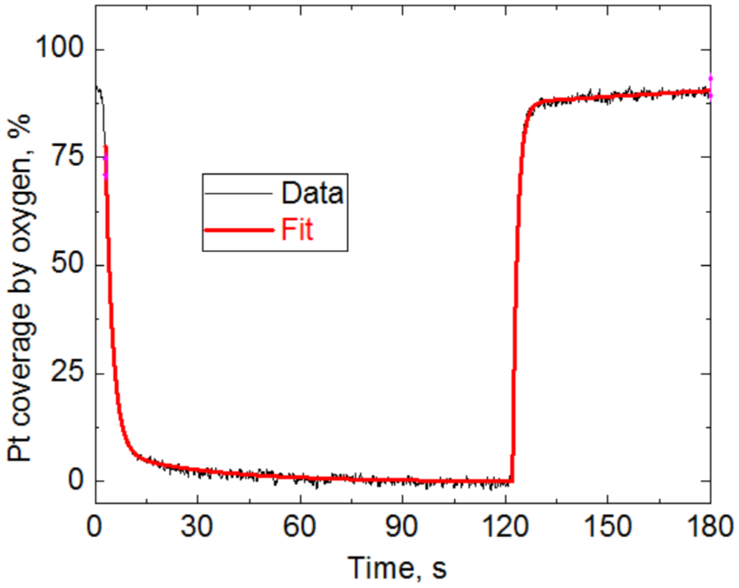		
**Parameter**	**Value**	**Error**
C_0	0.072	0.004
t_0	2.413	0.027
τ_1	2.144	0.042
τ_2	28.42	2.773
C_1	0.125	0.002
t_1	2.075	0.015
τ_3	1.431	0.023
τ_4	223.8	21.93
Fit index, χ	1.313	

Coverage of platinum by oxygen, %	T = 150 °C	z = 0.0 mm from the inlet

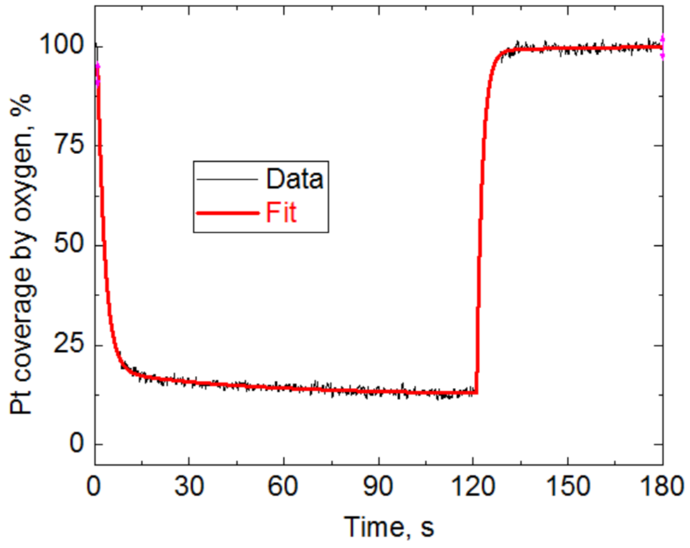

Parameter	Value	Error
C_0	0.072	0.003
t_0	0.834	0.019
τ_1	2.315	0.035
τ_2	44.99	5.592
C_1	0.012	0.003
t_1	1.046	0.016
τ_3	1.792	0.027
τ_4	36.08	13.62
Fit index, χ	1.090	

**Coverage of platinum by oxygen, %**	**T = 150 °C**	**z = 0.4 mm from the inlet**

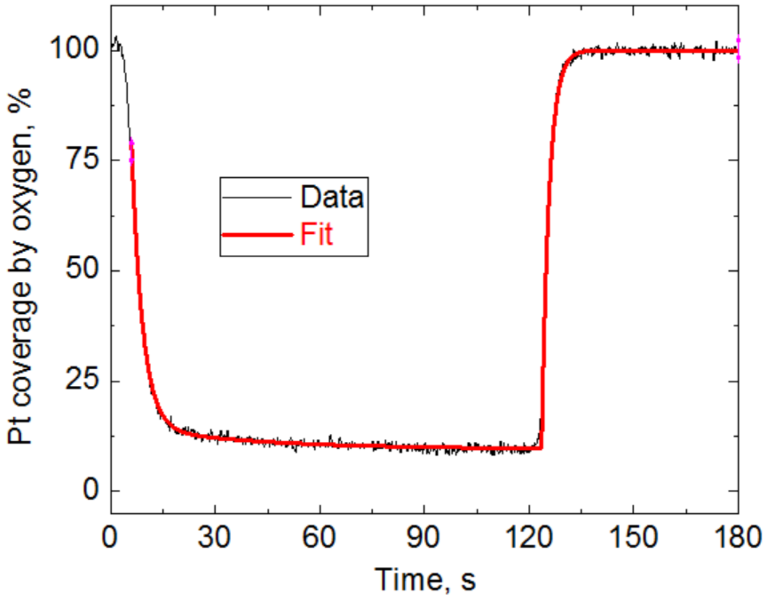

**Parameter**	**Value**	**Error**
C_0	0.056	0.005
t_0	5.090	0.042
τ_1	3.185	0.068
τ_2	35.32	6.568
C_1	0.000	-
t_1	3.616	0.019
τ_3	2.129	0.026
τ_4	-	-
Fit index, χ	1.462	

Coverage of platinum by oxygen, %	T = 150 °C	z = 0.8 mm from the inlet

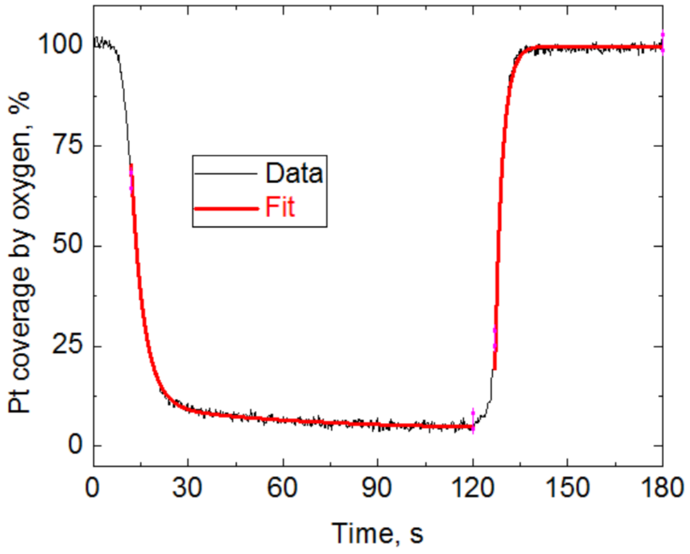

Parameter	Value	Error
C_0	0.070	0.003
t_0	10.39	0.049
τ_1	4.008	0.073
τ_2	51.89	10.35
C_1	0.000	-
t_1	6.802	0.023
τ_3	2.320	0.031
τ_4	-	-
Fit index, χ	1.503	
